# Crystalline Dion-Jacobson 2D Layered Sn-Based Perovskites for Field-Effect Transistors

**DOI:** 10.1021/jacs.5c20756

**Published:** 2026-03-20

**Authors:** Zhitian Ling, Shuanglong Wang, Arup Sarkar, Chongyao Li, Lei Gao, Ruyan Zhao, Dag W. Breiby, Dwight S. Seferos, Edward H. Sargent, Hai I. Wang, Mischa Bonn, Denis Andrienko, Paul W. M. Blom, Wojciech Pisula, Tomasz Marszalek

**Affiliations:** † 28308 Max Planck Institute for Polymer Research, Ackermannweg 10, Mainz 55128, Germany; ‡ Department of Applied Physics, 26680 the Hong Kong Polytechnic University, Hong Kong SAR 999077, P. R. China; § Department of Chemistry, 7938 University of Toronto, 80 St. George Street, Toronto M5S 3H6, Ontario, Canada; ∥ Department of Physics, Norwegian University of Science and Technology (NTNU), Høgskoleringen 5, Trondheim 7491, Norway; ⊥ Department of Electrical and Computer Engineering, 7938 University of Toronto, 35 St. George Street, Toronto M5S 3G4, Ontario, Canada; # Department of Molecular Physics, Faculty of Chemistry, Lodz University of Technology, Zeromskiego 116, Lodz 90-924, Poland

## Abstract

Dion-Jacobson (DJ) phase perovskites are increasingly recognized as potential semiconductor materials for electronic applications. However, the restricted coordination between the organic diammonium cations and the inorganic framework inhibits their self-assembly into efficient DJ Sn-based perovskites during solution processing. This study introduces solvent vapor assisted drop casting (SVAD) as a novel processing method to favor the organization into highly crystalline DJ perovskite films. The key aspect of the processing is the extended crystallization time in solvent vapor at elevated temperature. The processing study was conducted on four DJ perovskites that varied in the rigidity and symmetry of their A-cations to demonstrate the universal applicability of SVAD for this category of semiconductors and to establish correlations between structure and properties. The SVAD films exhibit markedly improved crystallinity and morphology compared to spin-coated samples resulting in significantly increased local and device charge carrier mobilities. DJ perovskites with rigid and symmetric A-cations form a planar [SnI_6_]^4–^ octahedral framework that favors structural order and charge carrier transport. This work paves the way for the solution processing of highly ordered DJ Sn-based perovskites for their implementation in electronic applications.

## Graphical abstract

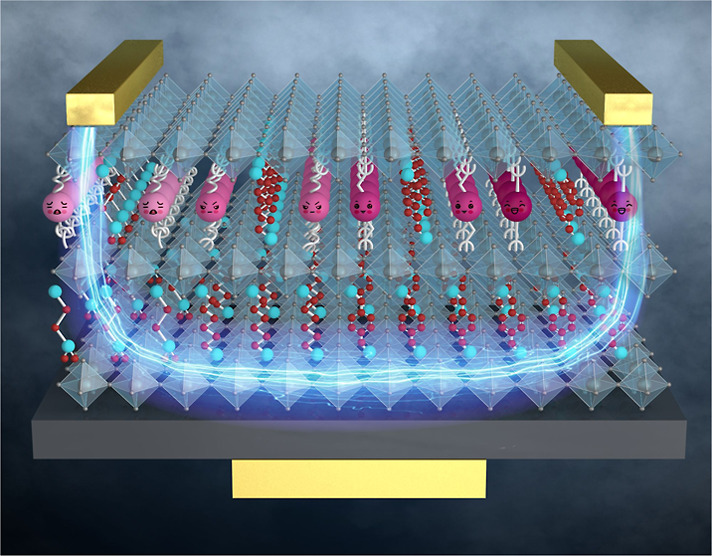

## Introduction

Tin-based halide perovskites have attracted increasing interest for applications in optoelectronic devices due to their low toxicity and high intrinsic mobility.^1,2^ However, three-dimensional Sn-based perovskites suffer from poor operational stability owing to the facile oxidation of Sn^2+^, rapid crystallization, and high sensitivity to moisture and oxygen.^3^ To address these challenges, large hydrophobic organic cations are introduced to form two-dimensional (2D) layered Sn perovskites with corner-sharing octahedral frameworks, which significantly enhance environmental stability.^4^ The 2D Sn perovskites are commonly classified into Ruddlesden–Popper (RP) and Dion-Jacobson (DJ) phases depending on the nature of the organic spacers.^5–8^ Bivalent ligands in DJ perovskites create closely packed inorganic layers without van der Waals gaps, unlike RP structures with monovalent cations, thereby promoting superior out-of-plane charge carrier transport.^9–13^ Recent studies have comparable charge carrier transport in out-of-plane and in-plane directions for DJ perovskites, indicating their potential as semiconductors for electronic applications.^14,15^

Although DJ perovskites are promising, their processing into crystalline films remains challenging and far from satisfactory for efficient charge carrier transport in field-effect transistors (FETs). DJ perovskites consist of divalent organic spacer A-cations that bridge adjacent inorganic octahedral layers via two terminal ammonium groups, which makes lattice matching and bonding chemistry more demanding compared to the simpler RP phases with incorporated monovalent spacers.^16,17^ Since Sn-based octahedral layers are already less robust and more distorted due to the stereoactive lone pair of Sn^2+^ in comparison to Pb^2+^, the requirements for structure integrity are more crucial for Sn-based perovskites.^18,19^ Consequently, the majority of recent studies on Sn-based DJ perovskites have focused on quasi-2D architectures, which have been particularly successful in photovoltaic applications but inevitably involve structural and phase heterogeneity.^20,21^ In contrast, investigations on pure 2D DJ Sn-based perovskites remain limited, despite their importance as structurally well-defined platforms for charge transport studies in FETs. Up to now, only a very limited number of DJ spacer cations have demonstrated compatibility with Sn-based perovskites, as outlined in the following section. For instance, Ji et al. employed the symmetric divalent 1,4-butanediammonium (BDA) spacer cation for the construction of DJ perovskites and the corresponding FETs showed charge carrier mobility of 0.58 cm^2^ V^–1^ s^–1^.^22^ Qiu et al. improved the device mobility for this DJ perovskite to 1.6 cm^2^ V^–1^ s^–1^ by introducing ammonium salt interlayers.^23^ More recently, an odd–even effect in lattice formation and charge transport was observed for a 2D Sn-based DJ perovskite with symmetric linear diammonium spacers of varying chain lengths.^24^ Within this series, only BDASnI_4_ exhibited a crystalline well-developed layered structure with comparatively high FET mobility, highlighting the limited spacer compatibility in pure 2D Sn-based DJ systems. Regarding the asymmetric DJ cations, Park et al. incorporated 3-(aminomethyl)­piperidinium (3AMP) and 4-(aminomethyl)­piperidinium (4AMP) as diammonium organic spacers in the Sn-based DJ perovskite. Due to the poor crystallinity of the spin-coated films the devices revealed a poor field-effect mobility of only 0.032 and 0.001 cm^2^ V^–1^ s^–1^ for 3AMP and 4AMP, respectively.^25^ In more recent work by Liao and co-workers, a device mobility of 0.57 cm^2^ V^–1^ s^–1^ was demonstrated for a (4AMP)­SnI_4_ single crystal.^26^ However, the use of single crystals makes the device fabrication difficult and complex, which presents a significant challenge for the perovskite integration in electronic devices.

To demonstrate the fundamental feasibility of DJ Sn-based perovskites in FETs, a novel approach has been developed for the efficient and straightforward solution deposition of uniform and highly crystalline DJ perovskite thin films. This method is based on thermal annealing of a drying film under a controlled vapor atmosphere, so-called solvent vapor assisted drop casting (SVAD) and is applicable to a broad range of organic A-cations, including asymmetric and symmetric building blocks of different rigidity. While spin-coating yields poorly ordered DJ perovskite films with low device performance, SVAD effectively prolongs the crystallization process during film drying ensuring sufficient time for the assembly of the organic spacer cations together with the inorganic [SnI_6_]^4–^ octahedron framework. As a result of this process, the activation barrier for crystallization is efficiently overcome resulting in high crystallinity and significantly improved charge carrier transport in comparison to the spin-coated films, as confirmed by optical pump-terahertz probe (OPTP) spectroscopy. FETs based on the SVAD processed DJ perovskites exhibit superior device behavior with pronounced gate modulation, high charge carrier mobility, minimized dual-sweep hysteresis as well as substantially enhanced bias stress stability. The processing method allows a simple fabrication of phase-pure DJ Sn-based perovskite films with symmetric and asymmetric A-cations for high-performance and reliable electronic devices.

## Results and Discussion

To prove the versatility of SVAD, which is discussed later in detail, four DJ-type organic spacer cations, *N*,*N*-dimethyl-1,3-propane diammonium (DMePDA), hexane-1,6-diammonium (HDA), 1,4-cyclohexyldimethylammonium (CDMA), and 1,4-phenyldimethylammonium (PDMA), with different symmetry and molecular rigidity were selected for the synthesis of the 2D layered Sn-based perovskites (Figure 1a). The role of the organic cations on the electronic structure is investigated by density functional theory (DFT) calculations. The optimized geometries derived from periodic DFT calculations are shown in Figure 1b. The organic spacers are modeled within the inorganic octahedra in such a way that minimizes the steric hindrance. Out of the four organic spacers, only DMePDA has an asymmetric structure, which was placed in an antiparallel arrangement with respect to the terminal NH_3_ and NH­(CH_3_)_2_ groups in order to minimize steric hindrance within the alkyl chains as well as in the inorganic–organic interaction.^27–29^ For all four perovskites, the corner-sharing [SnI_6_]^4–^ octahedral framework forms layers that are parallel to the sample substrate, here modeled as being in the *b*-*c* plane of the unit cell. Further perspectives of the cells are shown in Figures S1–S4 (Supporting Information). The optimized corner-sharing octahedral structures are employed for the simulation of Grazing Incidence Wide-Angle X-ray Scattering (GIWAXS) based on the DFT-optimized DJ perovskite structures, which is in agreement with the experimental data in Figure S5. The interlayer spacing between the inorganic octahedral layers is similar in (CDMA)­SnI_4_ and (PDMA)­SnI_4_ with 12.2 Å and 12.3 Å, respectively, and decreases to 10.3 Å and 11.8 Å for (DMePDA)­SnI_4_ and (HDA)­SnI_4_, respectively. Among the four structures, (DMePDA)­SnI_4_ and (HDA)­SnI_4_ arrange in a triclinic lattice configuration (where *a* ≠ *b* ≠ *c*, and α ≠ β ≠ γ), with one lattice angle deviating notably from 90°. Specifically, the angle α is 75.7° for (HDA)­SnI_4_ indicating heavier structure distortion. On the other hand, (CDMA)­SnI_4_ and (PDMA)­SnI_4_ form a crystal with a monoclinic space group (*a* ≠ *b* ≠ *c*, with α = γ = 90° and β ≠ 90°). Compared with the previous two perovskites, the monoclinic lattice exhibits a less tilted structure which offers more efficient pathways for the charge carrier transport.

**Figure 1:**
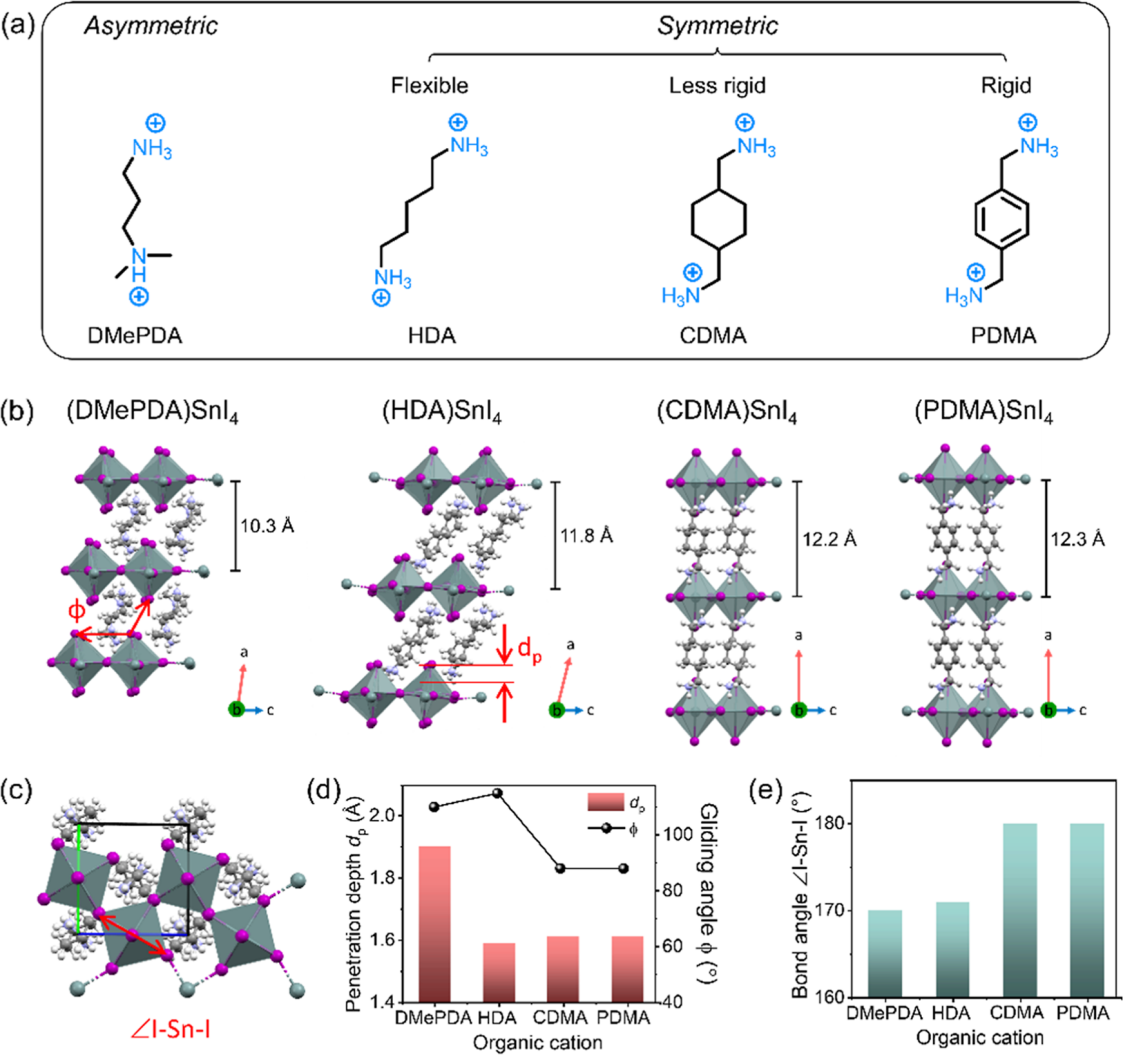
(a) Chemical structures of studied DJ-type organic cations: DMePDA; HDA; CDMA and PDMA, respectively. (b) DFT-D3 optimized crystal structures of (DMePDA)­SnI_4_, (HDA)­SnI_4_, (CDMA)­SnI_4_, (PDMA)­SnI_4_. The distance between the adjacent inorganic layers is shown for each structure. Red insert defines the *d*_p_ of the organic cations and ϕ of adjacent octahedral layers. (c) Definition of the bond angle ∠I–Sn–I in the octahedra. (d) Corresponding d_p_ and Φ values as well as (e) bond angles ∠I–Sn–I for the studied DJ perovskites.

As discussed previously, the length and orientation of the organic spacers influence the structure and interlayer distance between the [SnI_6_]^4–^ octahedron network. To quantify the structural distortion and interlayer spacing, three parameters are introduced. The penetration depth (*d*_p_), which measures the extent to which the organic cations penetrate toward the [SnI_6_]^4–^ octahedral framework, and the gliding angle (ϕ), representing the interplanar angle between successive [SnI_6_]^4–^ octahedral frameworks, are illustrated in Figure 1d. The third parameter is the bond angle ∠I–Sn–I that corresponds to the distortion of the octahedron, as demonstrated in Figure 1e. A *d*_p_ value of 1.5–1.6 Å is determined for the symmetric (HDA)­SnI_4_, (CDMA)­SnI_4_ and (PDMA)­SnI_4_, while for the asymmetric (DMePDA)­SnI_4_ the parameter increases to 1.7 Å and 1.9 Å for the terminal groups NHMe_2_ and NH_3_, respectively. The two *d*_p_ values for (DMePDA)­SnI_4_ indicate that the two sides of the asymmetric organic spacer penetrate into the inorganic sheet at different distances, which intensifies the distortion of the octahedral layer. The ϕ parameter rises from 88° for (CDMA)­SnI_4_ and (PDMA)­SnI_4_ to 110° and 115° for (DMePDA)­SnI_4_ and (HDA)­SnI_4_, respectively. The significant deviation in ϕ from 90° observed for (DMePDA)­SnI_4_ and (HDA)­SnI_4_ implies structural tilt which is potentially due to lower rigidity of the organic cations compared to the other two. The ∠I–Sn–I bond angle declines from 180° for (CDMA)­SnI_4_ and (PDMA)­SnI_4_ to 170° and 171° for (DMePDA)­SnI_4_ and (HDA)­SnI_4_, respectively. The distortion of the octahedron is related to larger *d*_p_ and ϕ values, which lead to an increase of the effective mass and finally reduction in charge carrier transport, as discussed later.

Electronic density-of-states (DOS) and band structure calculations were performed using the hybrid Heyd-Scuseria-Ernzerhof (HSE06) functional to gain insight into the electronic structure and the mobility of charge carriers. In Figure S6, the partial DOS (pDOS) plots reveal that the valence band is mainly composed of I 5p and Sn 5s orbitals, while the conduction band predominantly consists of Sn 5p and I 5p orbitals. Notably, the organic cations do not exert a direct influence on the electronic density-of-states for the four perovskites, as none of their bands approaches the Fermi level or the regions near the valence band maxima (VBM) and conduction band minima. However, the variation in symmetry and rigidity of the organic spacers results in differences in their arrangement toward the [SnI_6_]^4–^ octahedral framework affecting its planarity and the interlayer distance. These differences influence the electronic band structure of the DJ perovskites.

The density of states and electronic band structure of the four DJ perovskites were computed using the HSE06 hybrid functional which has been shown to predict accurate band gaps in solids.^30,31^ The computed direct band gaps of the perovskites are 1.70 eV, 1.85 eV, 1.70 and 1.74 eV for (DMePDA)­SnI_4_, (HDA)­SnI_4_, (CDMA)­SnI_4_, and (PDMA)­SnI_4_, respectively (as marked in Figures S7). To understand the charge transport properties and to determine the effective mass of the hole carriers *m*_h_^eff^ at the VBM, a polynomial fitting procedure is adopted. The structural parameters are summarized in Table S1 and show similar *m*_h_^eff^ for (CDMA)­SnI_4_ (−0.175 for Γ→*Z* direction) and (PDMA)­SnI_4_ (−0.182 for Γ→*Z* direction). The value increases for (DMePDA)­SnI_4_ and (HDA)­SnI_4_ to −0.413 and −0.341, respectively, in the Γ→*T* direction. The *m*_h_^eff^ has a negative sign because holes are considered in the valence band, which correspond to negative curvatures in the band structure.^32^ This increase is related to the flatter VBMs of (DMePDA)­SnI_4_ and (HDA)­SnI_4_ due to more distorted inorganic layers as compared to the more planar and ordered (CDMA)­SnI_4_ and (PDMA)­SnI_4_.

Following the elucidation of the role of the organic cations on the electronic structure using DFT calculations, the deposition of 2D layered DJ perovskite films was the subject of further study. Since DJ cations act as organic bridges connecting two adjacent [SnI_6_]^4–^ octahedral layers during crystallization the deposition method needs to facilitate the assembly of the organic and inorganic components into a well-ordered organization by controlling the crystallization rate over an adequately extended processing time. During spin-coating, the solvent evaporation rate is high resulting in kinetically nonequilibrium states of reduced crystallinity and diminished phase purity of the perovskite structure, as discussed later in detail. To address this issue, SVAD as a deposition process is developed for DJ perovskites and is illustrated in Figure 2a. During this procedure, the precursor solution is drop-cast on a heated silicon/silicon dioxide substrate at 100 °C and immediately covered. Under these conditions, the solution spreads spontaneously and uniformly over the entire substrate. After several minutes of solvent vapor treatment, the film starts to nucleate. Subsequently, the crystals grow with the color changing to dark indicating the formation of the perovskite phase. Once a dry and dark film is formed, the cover is removed, and the film is annealed at 100 °C for a further 10 min to evaporate any residual solvent. The detailed parameters of the SVAD process are provided in the Experimental section.

**Figure 2:**
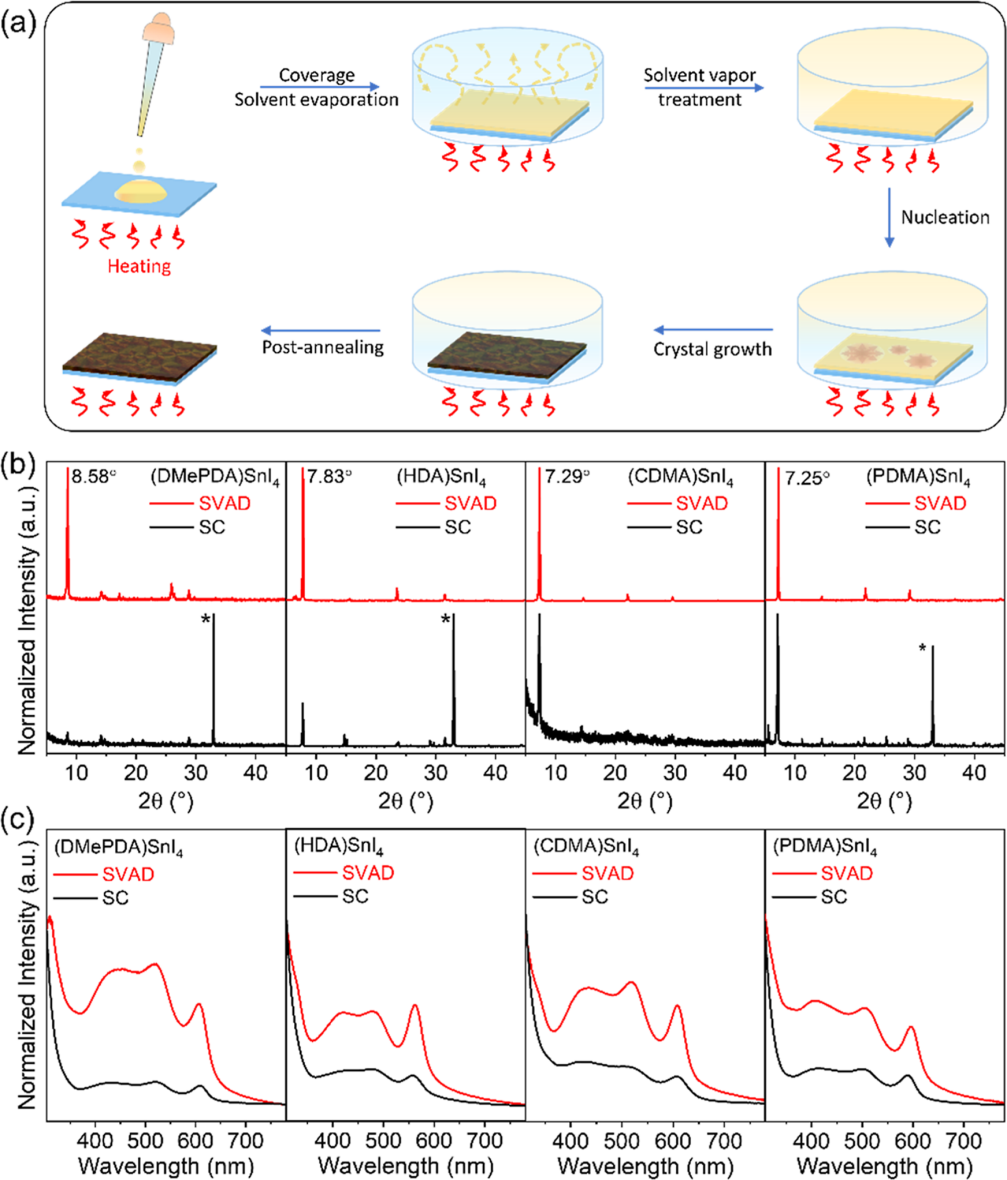
(a) Schematic illustration of the SVAD process for deposition of DJ-type 2D layered Sn-based perovskite thin films. (b) X-ray diffractograms and (c) absorption spectra of (DMePDA)­SnI_4_; (HDA)­SnI_4_; (CDMA)­SnI_4_; and (PDMA)­SnI_4_ films obtained by SVAD (red curve) and SC (black curve), respectively.

It should be emphasized that drop-cast films dried without the cover are nonuniform and reveal a high surface roughness, as shown in Figure S8a. In contrast, the SVAD significantly improves the film homogeneity and crystallinity (Figure S8b). Interestingly, the addition of ethanol as an additive to the precursor solution enhances the film coverage due to a uniform solution spreading over the substrate (Figure S8c). More importantly, the thickness of the SVAD films can be precisely controlled by adjusting the deposited volume of the precursor solution. Figure S9 demonstrates a significantly higher variation in film coverage for drop-casting in comparison to SVAD.

When the solution is drop-cast on the heated substrate, the uneven heat distribution across the deposited drop leads to a more rapid concentration increase at the edge compared to the center. Typically, this inhomogeneous distribution of the concentration leads to a coffee-ring effect as the capillary flow replenishes the evaporating liquid from the edge with liquid from the center resulting in low coverage and inconsistent thickness of the dry film.^33^ During SVAD, evaporation occurs gradually within a confined space. The saturated vapor environment, which is quickly formed after coverage, improves the wettability of the solution over the substrate by reducing the difference of the surface tension between the solid/vapor and solid/liquid interfaces. This phenomenon allows a full film coverage of 3 cm^2^ even with a small droplet volume of 20 μL. The extended evaporation time enables the precursors to assemble into an ordered perovskite structure additionally promoting film crystallinity. The SVAD process efficiently employs a very low precursor concentration of only 0.005 M, in contrast to the significant waste of the highly concentrated precursor solution during spin-coating.

The crystal structures of the SVAD and spin-coated (SC) films were characterized by X-ray diffraction (XRD) analysis. The film thickness of all DJ perovskites was kept constant at approximately 85 nm to allow a direct comparison of the width and intensity of the diffraction peaks. As shown in Figure 2b, films prepared by conventional SC exhibit less well-defined reflections and higher background levels. These findings suggest that only a small fraction of the 2D layered DJ perovskite phase is formed, additionally of poor crystallinity. The strong diffraction peak (marked as * in Figure 2b) at 2θ of 33° is attributed to the silicon wafer (200) and indicates a low surface coverage of the films after spin-coating, as discussed later. In contrast, films deposited by SVAD show distinct diffractograms with high intensity diffraction peaks that are characteristic for a 2D layered perovskite structure. The interlayer distances, calculated from the strong diffraction peaks located at 8.58°, 7.83°, 7.29°, and 7.25° for (DMePDA)­SnI_4_, (HDA)­SnI_4_, (CDMA)­SnI_4_, and (PDMA)­SnI_4_, are 10.4 Å, 11.4 Å, 12.1 Å, and 12.3 Å, respectively. These values derived from the XRD measurements align closely with the theoretical distances from the DFT calculations shown in Figure 1b, supporting the formation of the DJ perovskite structure.^23–25^

To further analyze the effect of the deposition method on the crystal size, the full width at half-maximum (fwhm) and the coherence length (CL) were calculated for the prominent 100 diffraction peak and are summarized in Table S2. Despite the similarity in thickness, the SVAD deposited films reveal larger CLs than spin-coated layers, emphasizing the superior crystallinity in the out-of-plane direction. Films comprising symmetric organic cations exhibit a higher long-range order compared to layers with the asymmetric building block DMePDA. Notably, the PDMA-based perovskite, with the most rigid cation structure, results in the highest CL of 85 nm, which nearly corresponds to the film thickness.

The improvement in film quality of the SVAD processed DJ-type perovskites is further confirmed by UV–vis absorption measurements. As shown in Figure 2c, and consistent with the XRD results, SVAD films exhibit similar absorption spectra with three markedly enhanced peaks in comparison to the spin-coated samples. The first peak, at the lowest wavelength, corresponds to the high-energy exciton transition level of the perovskite. The intermediate absorption feature is attributed to a long-lived exciton state, likely stabilized by the strong electrostatic field created by positively charged organic cations surrounding the negatively charged octahedral layers.^34^ The peak at the highest wavelength indicates intrinsic exciton absorption within the octahedral lattice. The higher intensity of these absorption peaks suggests improved order of the SVAD films. The optical band gap, derived from the sharp absorption edge of the third peak, is estimated at 1.78 eV for (DMePDA)­SnI_4_, 1.90 eV for (HDA)­SnI_4_, 1.77 eV for (CDMA)­SnI_4_, and 1.81 eV for (PDMA)­SnI_4_. These experimentally determined values are in good agreement with the computed data and the deviations are below 0.1 eV, supporting the internal consistency between experiment and theory. The corresponding photoluminescence (PL) spectra in Figure S10 exhibit strong emission peaks at 641, 595, 652, and 628 nm for the perovskites with DMePDA, HDA, CDMA and PDMA, respectively. In contrast, the spin-coated DJ perovskites exhibit broader and slightly shifted PL peaks compared to the SVAD films, which may be attributed to defects arising from their lower molecular order. The difference in optical band gap and emission peak of the four perovskites is related to the different energy band structures induced by variations in the crystal structure, as discussed earlier.

The morphology of the SC and SVAD processed DJ perovskite films with the four organic cations is further investigated by optical microscope (OM). All SVAD films demonstrate large domains with sizes over 100 μm in the OM images of Figure 3a. Additionally, polarized OM (POM) in Figure 3b discloses that the morphology of the SVAD films consists of highly birefringent spherulites. The Maltese cross in the POM images suggests radially oriented crystallized structures in the spherulitic domains of optical anisotropy, as shown in Figure S11.^35–37^ In contrast, only feature-less OM and dark POM images are acquired for the SC samples, as shown in Figure S12, indicating much smaller domains and lower crystallinity compared to the SVAD films.

**Figure 3:**
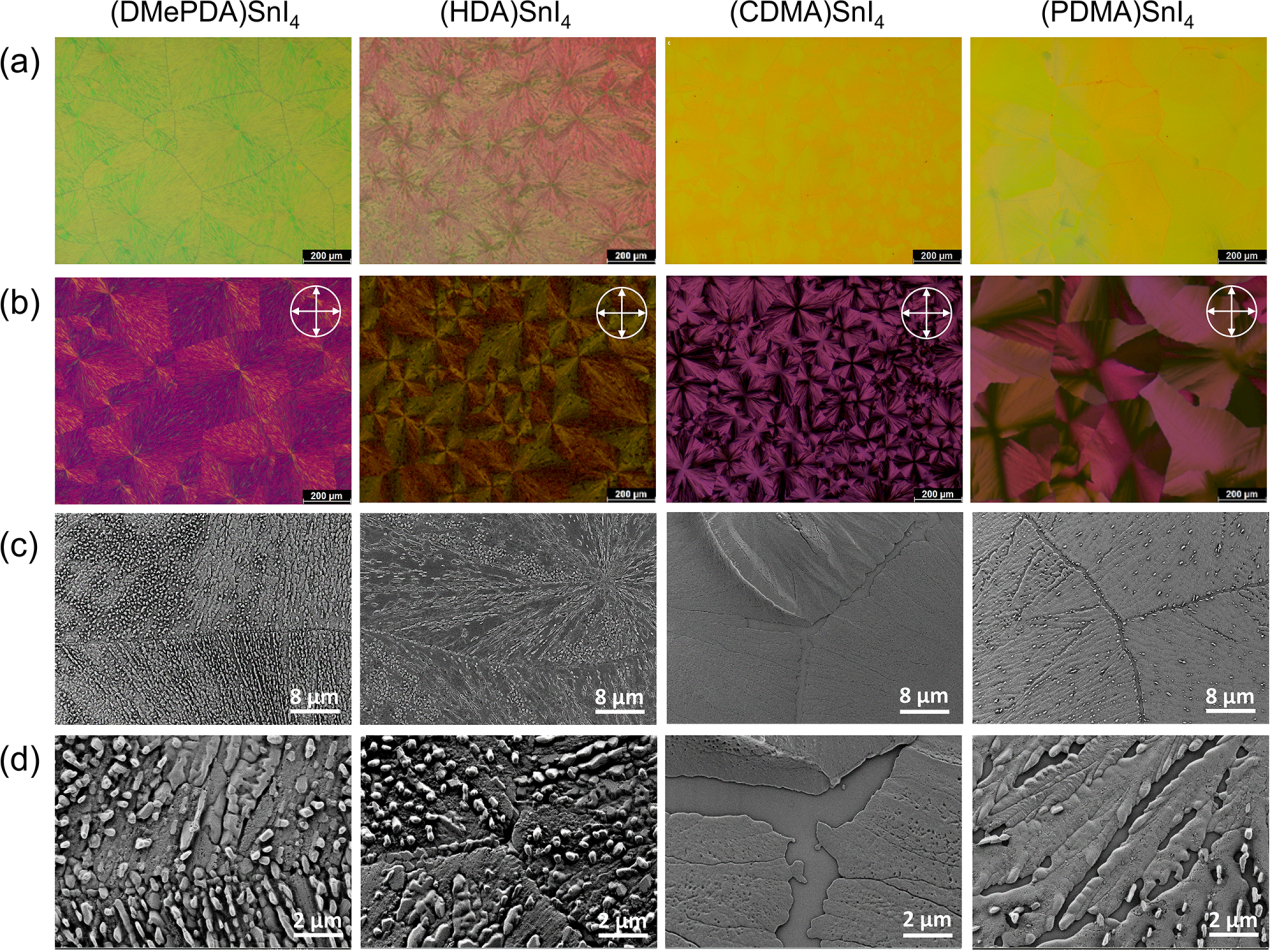
Morphology characterization of (DMePDA)­SnI_4_, (HDA)­SnI_4_, (CDMA)­SnI_4_ and (PDMA)­SnI_4_ SVAD films using (a) optical and (b) polarized optical microscope, (c) scanning electron microscope (SEM) with corresponding (d) zoom-in.

The morphology of the SVAD films is further investigated using scanning electron microscope (SEM). As shown in the SEM images in Figure 3c,d, all SVAD films form large spherulites composed of radially grown crystals. In contrast, the SC samples exhibit numerous small domains and pin holes, as depicted in Figures S13 and S14, which are obstacles for an efficient charge carrier transport.

To assess the charge carrier transport properties of the SVAD processed perovskites, OPTP spectroscopy measurements were carried out. Due to the transient nature of the terahertz (THz) pulse (∼1–2 ps duration), the THz probe reports a local charge carrier mobility in the spatial range of ∼10 nm. The pump-induced THz absorption -Δ*E*/*E* is proportional to the photoconductivity Δσ, which is also related to the product of the photogenerated carrier density and mobility. The quantum-yield-mobility product (φμ) is given as a function of pump–probe delay time, reaching the maxima within the delay time of 1 ps. Compared to the SC films, the SVAD deposited perovskites exhibit higher local mobility due to higher crystallinity as confirmed by XRD. The perovskite structure with the different A-cations also plays a role in the local mobility. The mobilities for the SVAD films of (CDMA)­SnI_4_ and (PDMA)­SnI_4_ are comparable with 3.2 cm^2^ V^–1^ s^–1^ and 3.4 cm^2^ V^–1^ s^–1^, respectively, as shown in Figure 4a. These values are higher in comparison to the mobility of 1.8 cm^2^ V^–1^ s^–1^ for (HDA)­SnI_4_ and of 1.1 cm^2^ V^–1^ s^–1^for (DMePDA)­SnI_4_. It should be noted that the SC films reveal a similar trend in mobility, which are all summarized in Table S3. The correlation between the local charge carrier mobility and perovskite structure can be explained by the DFT simulations. Compared to the rigid organic spacers CDMA and PDMA, the flexible cation HDA induces a more tilted octahedral structure in the perovskite. This structure distortion is even stronger for the asymmetric organic spacer DMePDA due to the unbalanced penetration depth between the two different end groups. This distortion of the inorganic [SnI_6_]^4–^ octahedron network results in large effective hole-mass values and thus to the low local mobilities.^38^

**Figure 4:**
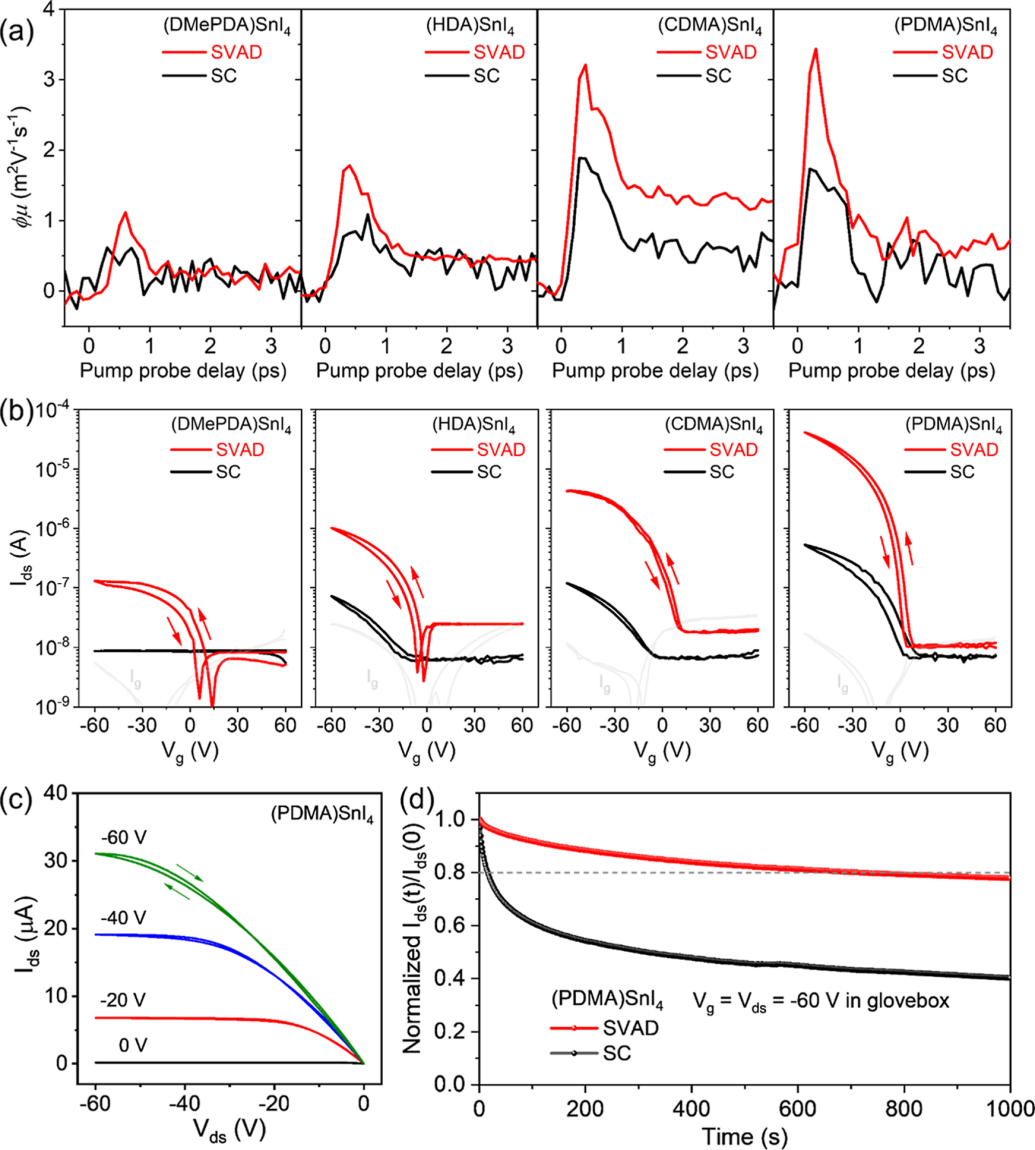
(a) Local charge carrier mobility φμ as a function of pump–probe delay time determined by optical pump-terahertz probe spectroscopy measurements. (b) FET transfer characteristics for SC and SVAD perovskite films based on DMePDA, HDA, CDMA and PDMA organic cations. (c) Output characteristics of SVAD (PDMA)­SnI_4_ FET. (d) Bias stress stability of (PDMA)­SnI_4_ FET under a constant bias of gate voltage (*V*_g_) = source-drain voltage (*V*_ds_) = −60 V.

The macroscopic charge carrier transport of the DJ perovskite films was investigated in bottom-gate top-contact FETs. The transfer and output characteristics were recorded at 295 K in glovebox under dark conditions with forward and reverse sweeps. For a fair comparison between SC and SVAD devices, the film thickness was controlled to be approximately 80 nm for a fully covered transistor channel. More details on the measurement procedure are given in the Experimental section. All electrical parameters are extracted from the transfer plots in forward direction. In Figure 4b, all SVAD devices exhibit a substantially improved performance including field-effect mobility (μ_FET_), threshold voltage (*V*_th_) and subthreshold swing (*SS*) compared to the SC films. The average device parameters are summarized in Table S4. In the case of the asymmetric (DMePDA)­SnI_4_, the SC FET does not show any field-effect response and any current flow over the whole *V*_*g*_ range due to the discontinuous film in the device channel shown in the SEM image in Figure S15a. The SVAD process significantly enhances the uniformity and order of the film resulting in a well-defined device performance with a field-effect mobility of 1.4 × 10^–3^ cm^2^ V^–1^ s^–1^. The perovskite with the softest symmetric spacer HDA also reveals an increase in device performance for the SVAD film from a low mobility of 2.5 × 10^–4^ cm^2^ V^–1^ s^–1^ and a high *SS* of 40.2 V dec^–1^ for the SC sample to 4.9 × 10^–3^ cm^2^ V^–1^ s^–1^ and 16.7 V dec^–1^. The charge carrier mobility of the SVAD film is further raised and the *SS* is decreased with increased rigidity of the organic cation and crystallinity to 0.02 cm^2^ V^–1^ s^–1^ for (CDMA)­SnI_4_ and 0.25 cm^2^ V^–1^ s^–1^ for (PDMA)­SnI_4_ demonstrating a 2 orders of magnitude higher mobility in comparison to the SC device. The discrepancy between the local mobility determined by OPTP and the macroscopic transistor mobility can be rationalized by the grain boundaries which are known to suppress charge carrier transport compared to transport within single crystalline domains.^39^ The SEM analysis reveals that SC films consist of substantially smaller domains than SVAD processed samples, leading to a higher density of grain boundaries. This structural difference is expected to severely limit long-range charge transport in FETs, consistent with the reduced mobility observed for SC devices. Besides grain boundaries, additional device-related factors further contribute to the discrepancy between OPTP and FET mobilities. The *SS* values, extracted from the transfer characteristics as shown in Table S4, indicate a finite density of interfacial trap states at the dielectric/semiconductor interface, which can reduce the apparent field-effect mobility by trapping charge carriers during steady-state transport.^40^ Notably, the SC devices exhibit systematically larger *SS* values than the SVAD devices, indicating a higher density of interfacial trap states, which can be attributed to the inferior crystallinity of the SC films revealed by XRD analysis. Apart from enhanced device mobility and smaller *SS*, the SVAD FETs also show smaller *V*_th_, e.g. 7.3 V for (PDMA)­SnI_4_, which can be also associated with larger domain size and much higher coherence length indicating lower defect density. Additionally, the output characteristics of SVAD processed (PDMA)­SnI_4_ FET in Figure 4c disclose a distinctive linearity at low *V*_ds_ and current saturation at high *V*_ds_ in contrast to the S-shaped output plots of the SC film in Figure S15. The S-shaped output characteristics observed in the SC devices indicate a substantial contact resistance at the source–drain electrodes, which impedes charge injection and reduces μ_FET_.^41,42^ In contrast, the nearly linear output characteristics of the SVAD devices suggest a reduced charge injection barrier at the perovskite/electrode interface, which further contributes to their markedly improved device performance. As a summary, the improved FET performance of the SVAD devices arises from the combined effects of enhanced crystallinity, reduced grain boundary density, lower interfacial trap density, and improved charge injection at the electrode interfaces.

Finally, the operational stability of the (PDMA)­SnI_4_ device was investigated, which is a crucial factor for practical applications. The bias stress stability of (PDMA)­SnI_4_ FETs, fabricated by SC and SVAD, was evaluated in a nitrogen-filled glovebox under dark conditions. Figure 4d depicts the change in source-drain current (*I*_ds_) under constant bias of *V*_g_ = *V*_ds_ = −60 V over a period of 1000 s. The SC device exhibits a sharp decline in the normalized source-drain current (*I*_DS_(t)/*I*_DS_(0)) compared to the SVAD processed FET, which demonstrates significantly enhanced bias stability. Specifically, the decay times to reach 80% of the initial *I*_ds_ increased from 16 s for the SC device to 712 s for the SVAD device. Such improvement can be attributed to higher film crystallinity.

## Conclusion

Highly crystalline 2D Sn-based DJ perovskites with four different symmetric and asymmetric A-cations were fabricated by the SVAD process. The key processing steps of SVAD include the annealing of the wet perovskite film in saturated solvent vapor to extend the crystallization time at the high temperature. Compared to conventional SC, the SVAD films exhibit higher molecular order and improved morphology characterized by large, birefringent spherulitic domains. As a consequence, the SVAD films show significantly increased local and field-effect charge carrier mobilities together with enhanced operational stability of the device. Among the fabricated films, symmetric DJ perovskites demonstrate higher charge carrier mobilities than their asymmetric analogues and this performance improves with increased rigidity of the organic cations. DFT calculations indicate that cation design plays an important role in the organization of the [SnI_6_]^4–^ octahedron framework. Symmetric and rigid cations ensure distinct planarity of the octahedron layers resulting in pronounced charge carrier transport. This work addresses the challenges of fabricating 2D Sn-based perovskite FETs with different types of DJ cations, providing solid foundation for their implementation in electronic devices.

## Experimental Section

### Materials Synthesis

All chemical reagents and solvents were used without further purification. Tin­(II) iodide (SnI_2_, 99.999%), *N*,*N*-dimethylformamide (DMF, anhydrous, 99.8%), and ethanol were purchased from Sigma-Aldrich. Silicon wafers were obtained from Ossila. The DJ type organic spacer cations, *N*,*N*-dimethyl-1,3-propane diammonium iodide (DMePDAI_2_) and hexane-1,6-diammonium iodide (HDAI_2_) were purchased from Greatcell Solar Materials. 1,4-Phenyldimethylammonium iodide (PDMAI_2_) was synthesized in Prof. Dwight S. Seferos’s group. 1,4-Cyclohexyldimethylammonium iodide (CDMAI_2_) was purchased from Xi’an Polymer Baolaite Photoelectric Technology Corporation.

### 2D Perovskite Thin Film Preparation

The precursor solutions were prepared in a stoichiometric ratio of DJ cations and SnI_2_ 1:1 by dissolved in pure DMF solvent. The solution was stirred overnight at 40 °C in a glovebox and cooled to room temperature before deposition. Heavily p-doped bare Si/SiO_2_ wafers (1.5 × 2 cm) were used as substrates and cleaned in an ultrasonic bath by deionized water, acetone, and isopropyl alcohol and in the next step blown dry with N_2_. The substrates were treated by oxygen plasma for 3 min and then transferred into the glovebox. Before film fabrication, a 0.2 μm PTFE filter was used for the precursor solution. The as-prepared fresh precursor solution with 0.1 M was spin-coated at 1500 rpm and 60 s on the silicon wafer and annealed at 100 °C for 10 min. For SVAD, the precursor solution was first diluted to a concentration of 0.005 M. A droplet of 20 μL (mixed with 2 vol % of ethanol) was cast on the substrate, immediately covered and annealed at 100 °C for 10 min. The confined solvent vapor environment is established by a well-defined reaction chamber volume of approximately 42 cm^3^. After this time, the dry film was further annealed at 100 °C for 10 min without the cover.

### Device Fabrication and Measurement

Bottom-gate top-contact configuration was employed for the FET devices. The 300 nm SiO_2_ layer of the silicon wafers was adopted as a dielectric layer. After film preparation, the source and drain electrodes were deposited at a thickness of 50 nm by gold thermal evaporation through a shadow mask to construct 80 × 1000 μm (length × width) transistor channels. The device characterization was performed using a semiconductor parameter analyzer (Keithley 4200-SCS). The transfer and output characteristics were recorded in pulse mode. In the pulse mode, *V*_g_ was applied over a short pulse of 1 s. Charge carrier mobility of the FETs was extracted from the following equation: $\mu =\frac{2L}{WC_{i}}{\left(\frac{\partial \sqrt{I_{ds}}}{\partial V_{g}}\right)}^{2}$ , where *L*, *W*, and *C*_*i*_ are the channel length and width and the unit capacitance of the oxide dielectric, respectively. The interface trap density (*N*_*t*_) at the dielectric/perovskite interface from *SS* using the following equation: *SS* = $\frac{K_{b}T\ln10}{q}\left(1+\frac{q^{2}}{C_{i}}N_{t}\right)$ , with *q* the elementary charge; *K*_b_ the Boltzmann constant; and *T* the absolute temperature.

### Characterization

The thin film morphology was characterized by a Leica POM and Hitachi scanning electron microscope. The thickness of the DJ perovskite films was measured by a Bruker Tencor Profilometer. GIWAXS measurements were performed at the TU Dortmund DELTA Synchrotron. The beam size at a photon energy of 10 keV was 1.0 mm × 0.2 mm (width × height). Samples were irradiated just below the critical angle for total reflection with respect to the incoming X-ray beam (∼0.1°). The CL was calculated for selected reflections from the Scherrer equation. The film XRD diffractograms were recorded using a Rigaku D/MAX 2600 V with Cu Kα (λ = 1.5406 Å) radiation. The optical absorption spectra of the perovskite films were recorded using a PerkinElmer Lambda 900 UV/vis spectrophotometer, featuring an all-reflecting, double-monochromator optical system with holographic gratings in each monochromator for the UV–vis range. Data was collected from 300 to 800 nm with a dwell time of 0.1 s. PL spectra were recorded using a HORIBA Jobin-Yvon Fluorolog 3–22 Tau-3, equipped with a photomultiplier tube (PMT) as the detector, and FluorEssence software (version 3.9.0.1; Origin version 8.6001). The samples were under nitrogen flow to prevent degradation of the perovskite samples. All measurements were conducted at room temperature.

### DFT Calculation

All calculations were carried out in Vienna Ab initio Simulation Package (VASP) 6.4.2 version, employing the projector augmented wave (PAW) method.^43–46^ Geometry optimizations were performed for all perovskites using Perdew–Burke–Ernzerhof (PBE)^47^ functional with the van der Waals dispersion via Grimme’s D3 correction along with the Becke-Johnson (BJ) damping function.^48,49^ Structural relaxation was performed with complete cell optimization (ISIF = 3) using an energy cutoff at 520 eV with 2 × 3 × 3 (for PDMA and CDMA), 2 × 2 × 2 (for HDA) and 3 × 3 × 3 (for DMePDA) Monkhorst–Pack^50^ *k*-point grid to sample the Brillouin zone. The number of valence electrons for the elements were chosen as Sn(14), C(4), I(7), N(5) and H(1). During the electronic density-of-states and band structure calculations, the screened hybrid functional of Heyd, Scuseria, and Ernzerhof (HSE06)^31^ was used. For the partial DOS *k*-point mesh increased to 4 × 6 × 6 (for PDMA and CDMA), 4 × 4 × 4 (for HDA) and 4 × 5 × 4 (for DMePDA) centered around Γ-point. Furthermore, high-symmetry *k*-points were chosen during the band structure calculations from the seek-*k* path tool.^51^ The optimized structures, pDOS and band structure were visualized and plotted using VESTA and SUMO packages.^52,53^ Effective masses of the carriers (holes/electrons) were computed by the third-order polynomial fitting using the VASPKIT utility (a postprocessing package for VASP code).^54^

### GIWAXS Simulation

The GIWAXS simulations were carried out using the software SimDiffraction, designed to provide accurate calculated scattering patterns for textured polycrystalline thin films.^55^ Based on the molecular crystal structures specified in the CIF files generated by the theoretical modeling, the GIWAXS patterns were predicted, taking into account the slight preferred orientation of the crystallites.

### OPTP Spectroscopy

The THz spectrometer was driven by Ti/sapphire amplified pulsed laser system with the following output features: 800 nm central wavelength, duration of ∼50 fs and a repetition rate of 1 kHz. The THz field was generated by optical rectification in a ZnTe crystal. The bandwidth of the THz pulse was ∼2 THz. The pump light of 400 nm was generated by second harmonic generation in a BBO crystal. The time dependent mobility was calculated from $\frac{\sigma }{N_{abs,flim}}=\left(-\frac{\varepsilon_{0}c\left(n_{1}+n_{2}\right)}{d}\cdot \frac{\Delta E}{E}\right)\cdot \frac{d}{N_{abs}}=\varphi \mu$ , where *N*_abs_ is the absorbed sheet photon density, *n*_1_ = 1.96 is the THz refractive index of fused silica, *n*_2_ = 1 the refractive index of the air. *N*_abs_ is obtained as the product of the incident photon density and the absorption. *N*_abs,flim_ is the number of absorbed photons in the unit thickness.

## Supplementary Material



## Notes

Materials and methods; experimental details; DFT calculation details; and characterization data, including polarized optical photographs, SEM, GIWAXS, XRD data analysis, PL and transistors output characteristic (PDF)
