# Supplementary material for: Crystalline Dion-Jacobson 2D Layered Sn-Based Perovskites for Field-Effect Transistors
Source: J Am Chem Soc. 2026 Mar 20;148(12):12764–74. doi: 10.1021/jacs.5c20756 (PMC13047535; doi:10.1021/jacs.5c20756)
Supplement: Supplementary file 1 [file ja5c20756_si_001.pdf]

Supporting Information

Crystalline Dion-Jacobson 2D Layered Sn-Based Perovskites for Field-Effect Transistors

Zhitian Ling[1,#], Shuanglong Wang[2,#], Arup Sarkar[1,#], Chongyao Li[1], Lei Gao[1], Ruyan Zhao[3], Dag W. Breiby[4], Dwight S. Seferos[3], Edward H. Sargent[5], Hai I. Wang[1], Mischa Bonn[1], Denis Andrienko[1], Paul W.M. Blom[1], Wojciech Pisula[1,6*] and Tomasz Marszalek[1,6*]

[1]Max Planck Institute for Polymer Research, Ackermannweg 10, 55128 Mainz, Germany

[2]Department of Applied Physics, The Hong Kong Polytechnic University, Hong Kong SAR 999077, P.R. China.

[3]Department of Chemistry, University of Toronto, 80 St. George Street, Toronto, Ontario M5S 3H6, Canada

[4]Department of Physics, Norwegian University of Science and Technology (NTNU), Høgskoleringen 5, 7491 Trondheim, Norway

[5]Department of Electrical and Computer Engineering, University of Toronto, 35 St. George Street, Toronto, Ontario M5S 3G4, Canada

[6]Department of Molecular Physics, Faculty of Chemistry, Lodz University of Technology, Zeromskiego 116, 90-924 Lodz, Poland

[#]These authors contributed equally to this work.

[*]To whom correspondence should be addressed.

Email: *pisula@mpip-mainz.mpg.de, marszalek@mpip-mainz.mpg.de*

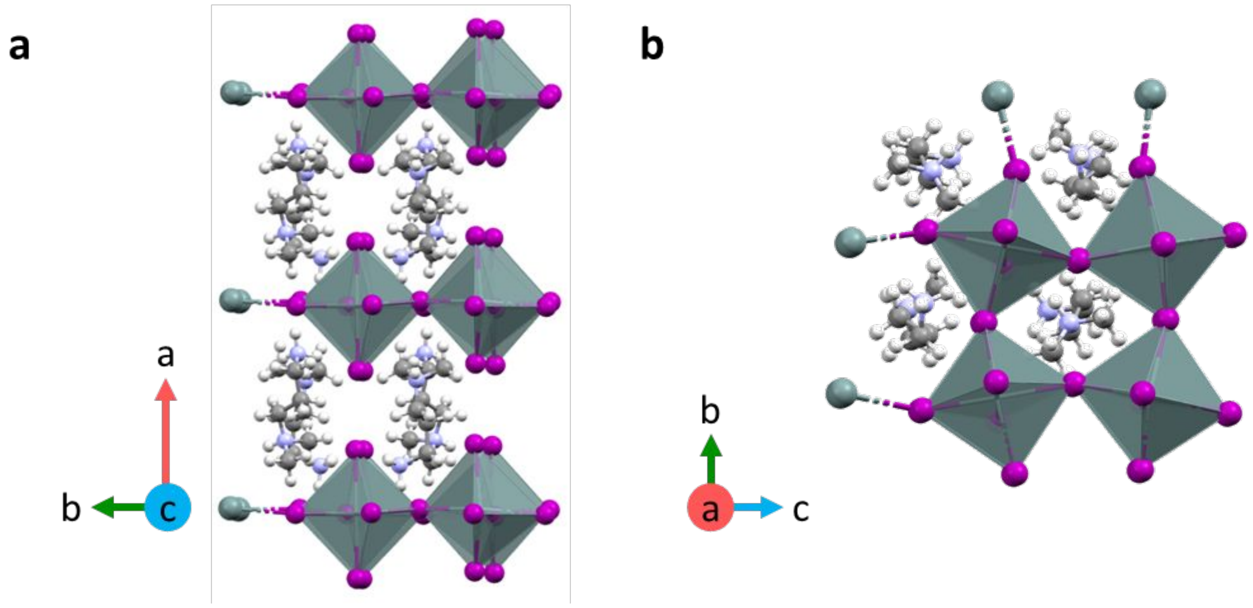

Figure S1. DFT-D3 optimized crystal structures of (DMePDA)SnI_4 with the cell view along the (a) **c** and (b) **a** axis.

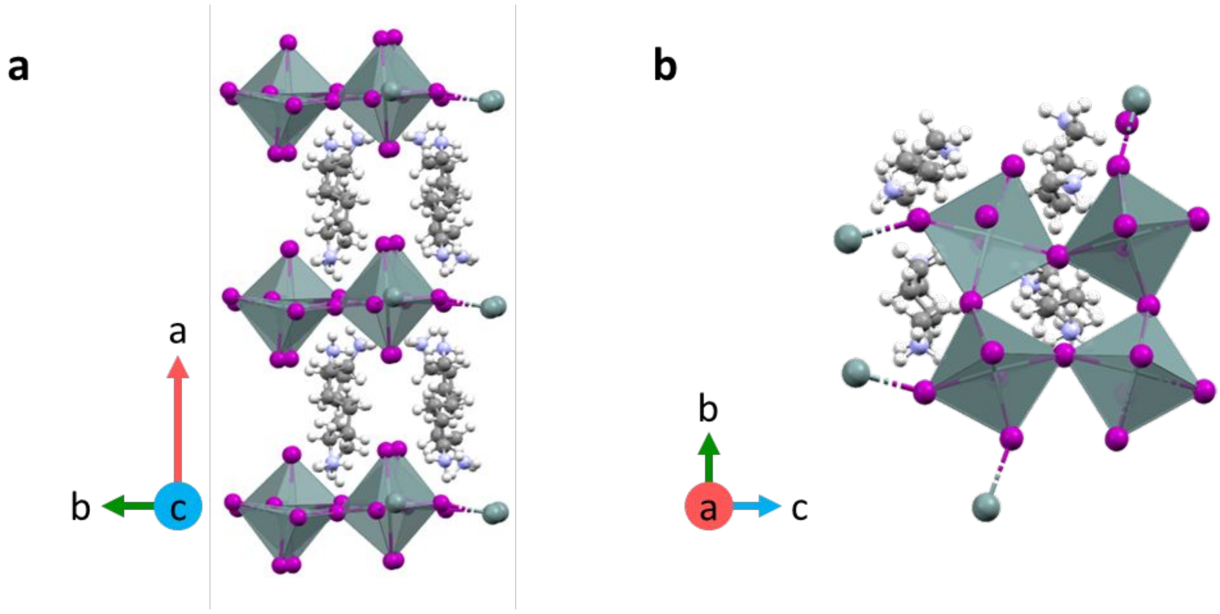

Figure S2. DFT-D3 optimized crystal structures of (HDA)SnI_4 with the cell view along the (a) **c** and (b) **a** axis.

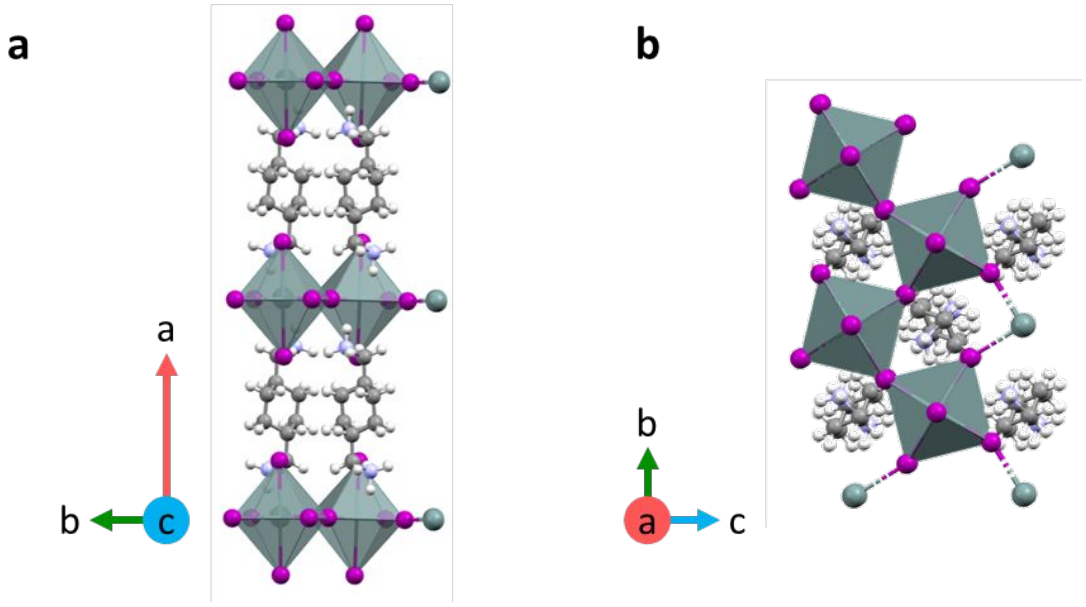

Figure S3. DFT-D3 optimized crystal structures of $(CDMA)SnI_4$ with the cell view along the (a) **c** and (b) **a** axis.

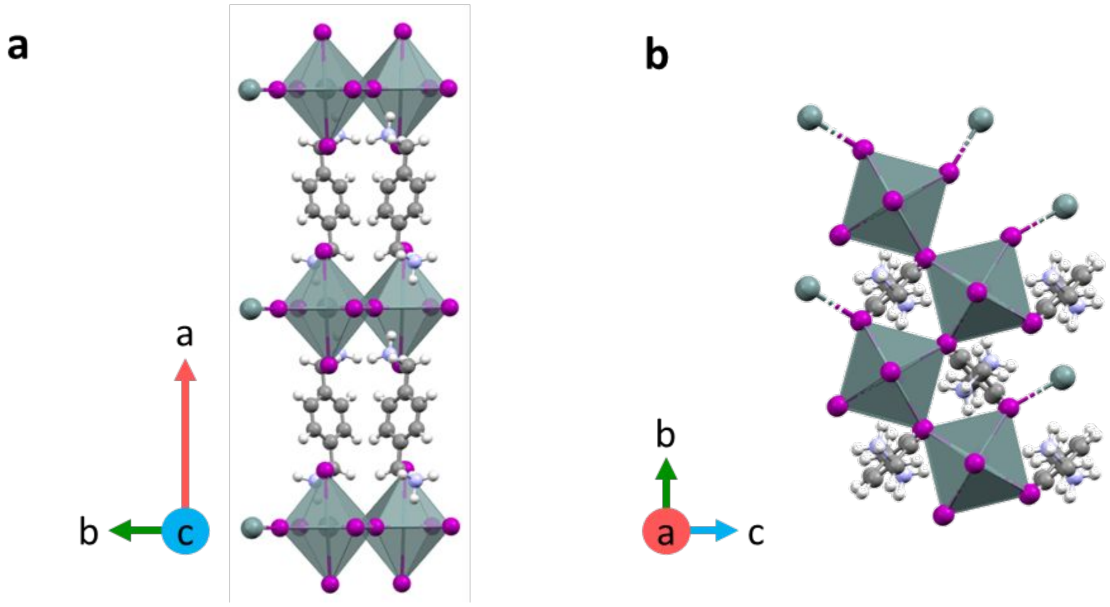

Figure S4. DFT-D3 optimized crystal structures of $(PDMA)SnI_4$ with the cell view along the (a) **c** and (b) **a** axis.

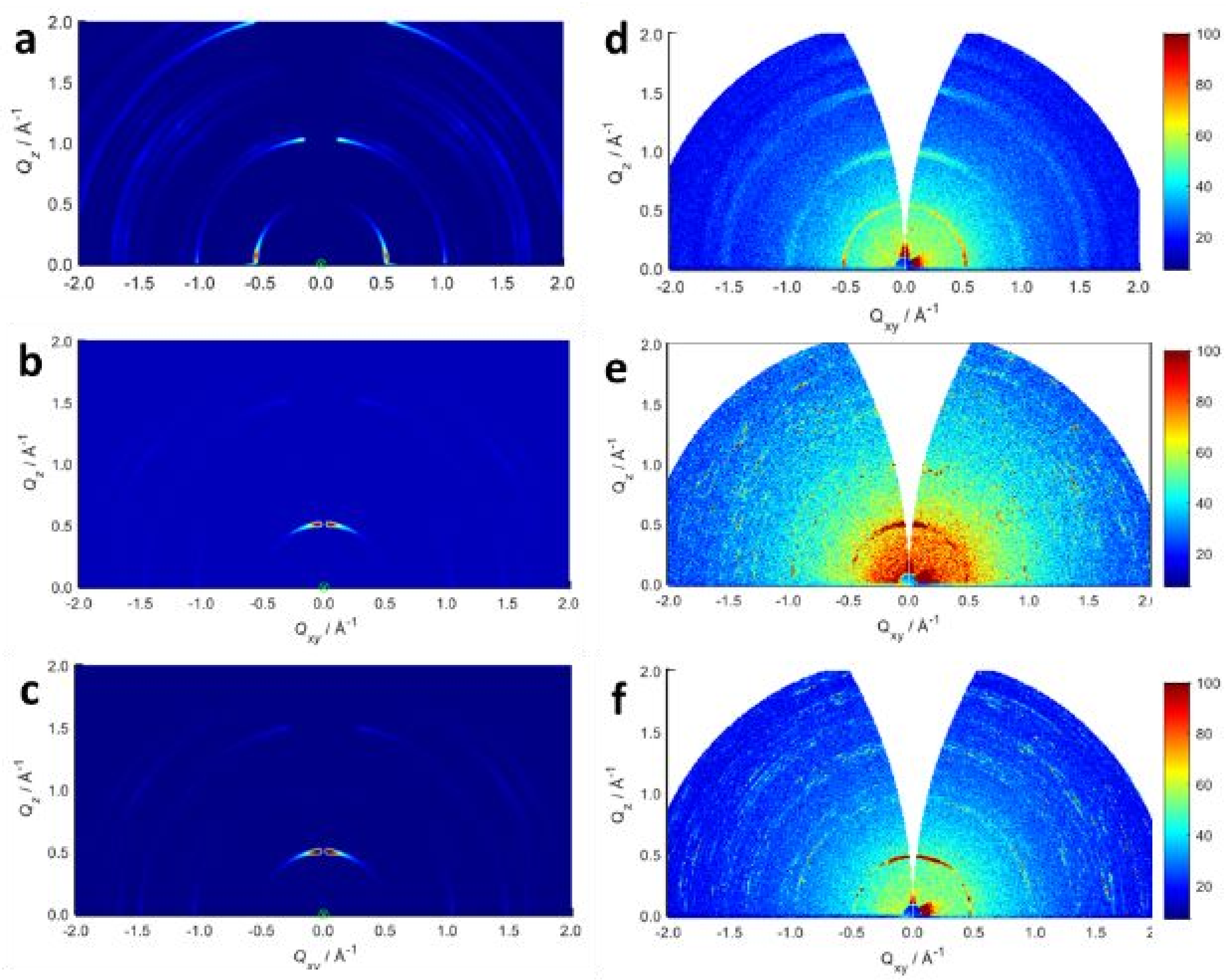

Figure S5. The simulated and experimental GIWAXS patterns for (a,d) (HDA)SnI_4, (b,e) (CDMA)SnI_4, and (c,f) (PDMA)SnI_4, respectively. The simulated pattens are presented in the left panel and the experimental results in the right panel.

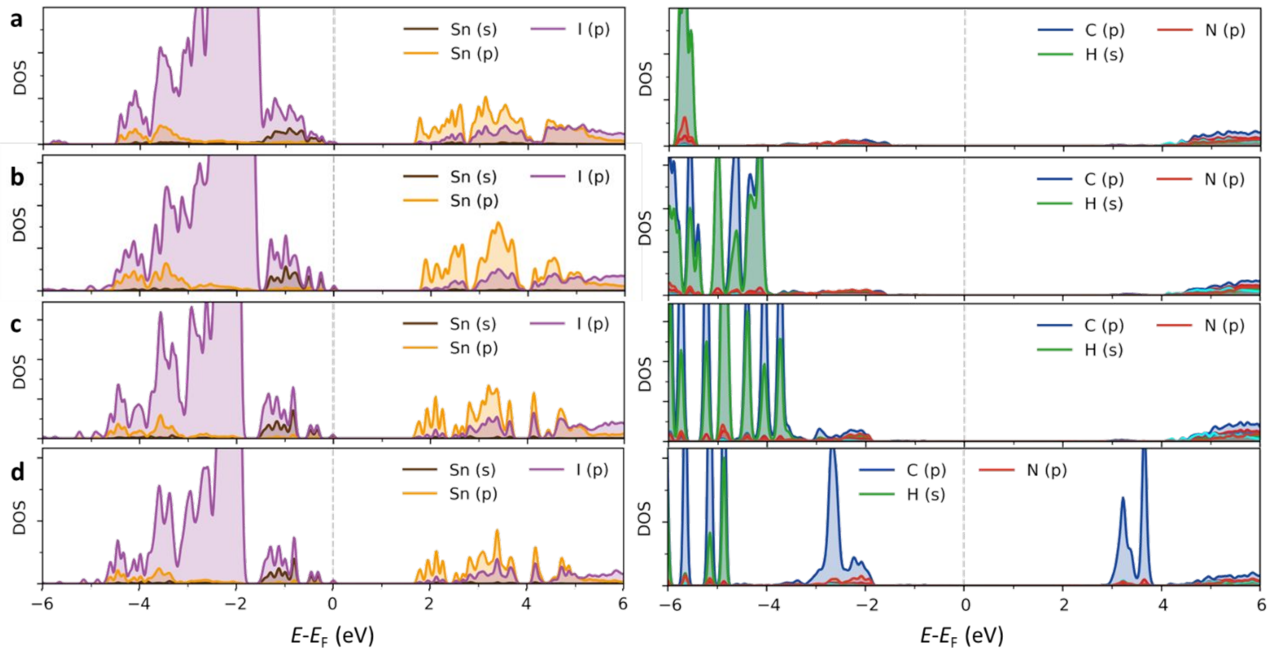

Figure S6. DFT computed partial density-of-states (pDOS) of (a) (DMePDA)SnI_4, (b) (HDA)SnI_4, (c) (CDMA)SnI_4 and (d) (PDMA)SnI_4. The pDOS are presented with the Sn and I contributions in the left panel and the organic spacer contribution in the right panel.

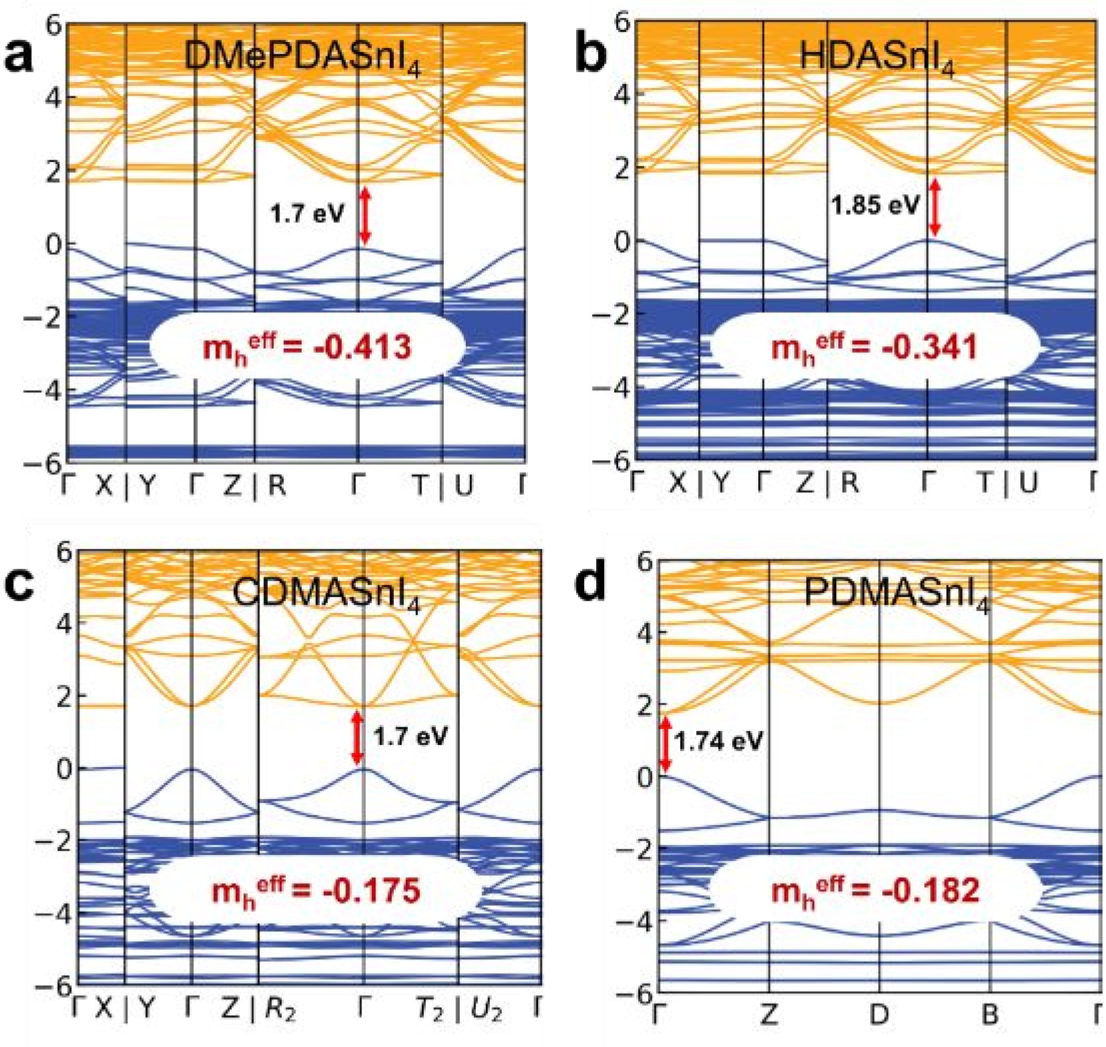

Figure S7. The band structure and the effective masses of (a) $(DMePDA)SnI_4$, (b) $(HDA)SnI_4$, (c) $(CDMA)SnI_4$ and (d) $(PDMA)SnI_4$ obtained from HSE06 functional. The effective masses for holes were calculated along the Γ→Z path direction for PDMA and CDMA and Γ→T path direction for HDA and DMePDA structures. The unit for the effective mass is given with respect to the rest mass of free electron ($m_0 = 9.11 \times 10^{-31}$ kg).

Table S1. Selected lattice and structural parameters for the DFT-D3 optimized structures

of the four perovskites.

Structures	Lattice parameters		Sn-I bond lengths	Bond angles
$(PDMA)SnI_4$	a = 12.323 Å<br>b = 8.516 Å<br>c = 8.530 Å	α = 90.0°<br>β = 91.2°<br>γ = 90.0°	3.198 Å<br>3.112 Å<br>3.113 Å	∠I-Sn-I = 180° (axial)<br>∠Sn-I-Sn = 151° (in plane)
$(CDMA)SnI_4$	a = 12.173 Å<br>b = 8.564 Å<br>c = 8.563 Å	α = 90.0°<br>β = 89.3°<br>γ = 90.0°	3.182 Å<br>3.123 Å<br>3.115 Å	∠I-Sn-I = 180° (axial)<br>∠Sn-I-Sn = 152° (in plane)
$(HDA)SnI_4$	a = 12.017 Å<br>b = 12.230 Å<br>c = 12.096 Å	α = 75.7°<br>β = 89.2°<br>γ = 89.0°	2.994 Å; 3.432 Å<br>3.075 Å; 3.186 Å<br>3.184 Å; 3.184 Å	∠I-Sn-I = 171° (axial)<br>∠Sn-I-Sn = 148°-153° (in plane)
$(DMePDA)SnI_4$	a = 12.378 Å<br>b = 10.447 Å<br>c = 12.362 Å	α = 81.5°<br>β = 90.2°<br>γ = 90.1°	3.234 Å; 3.193 Å<br>3.187 Å; 3.122 Å<br>3.049 Å; 3.191 Å	∠I-Sn-I = 170°-175° (axial)<br>∠Sn-I-Sn = 156°-160° (in plane)

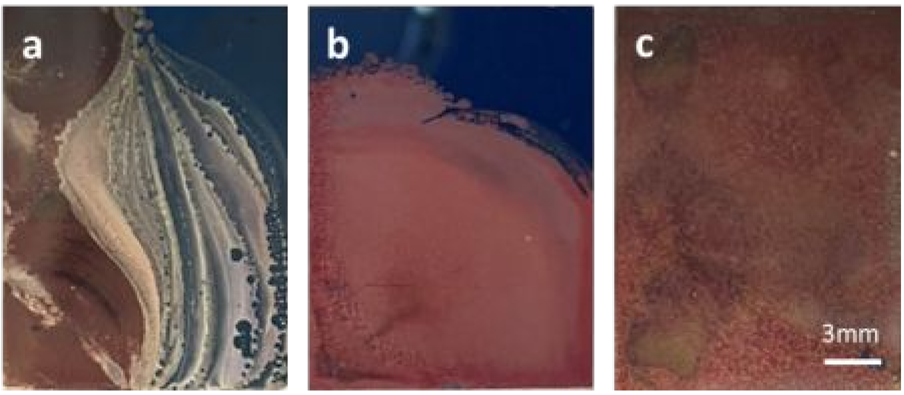

Figure S8. Microscopy images of the DJ-type 2D Sn-based perovskite $(PDMA)SnI_4$ deposited by (a) drop casting and (b) SVAD from a DMF solution; (c) SVAD from DMF solution mixed with ethanol as additive.

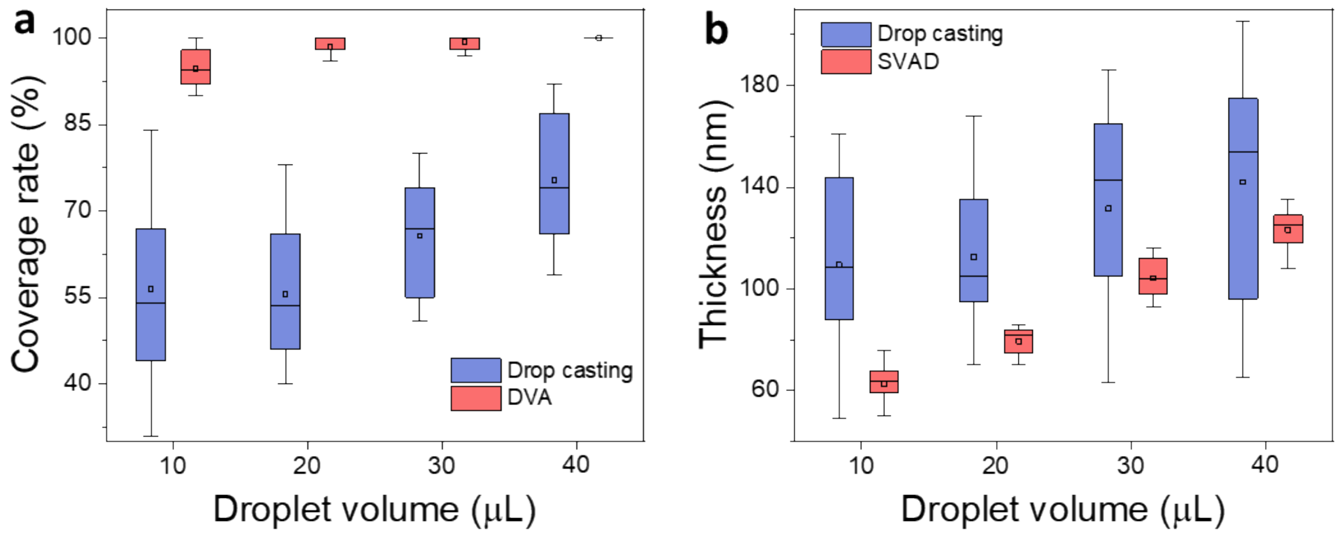

Figure S9. The relation between droplet volume and (a) coverage rate and (b) thickness of the dry film. Error bars are obtained from 10 samples.

Table S2. Calculated FWHM and CL from the *100* reflections.

		**(DMePDA)SnI$_4$**	**(HDA)SnI$_4$**	**(CDMA)SnI$_4$**	**(PDMA)SnI$_4$**
SVAD	FWHM (°)	0.16	0.13	0.13	0.09
	CL (nm)	50	63	63	85
SC	FWHM (°)	0.19	0.15	0.17	0.17
	CL (nm)	42	56	48	48

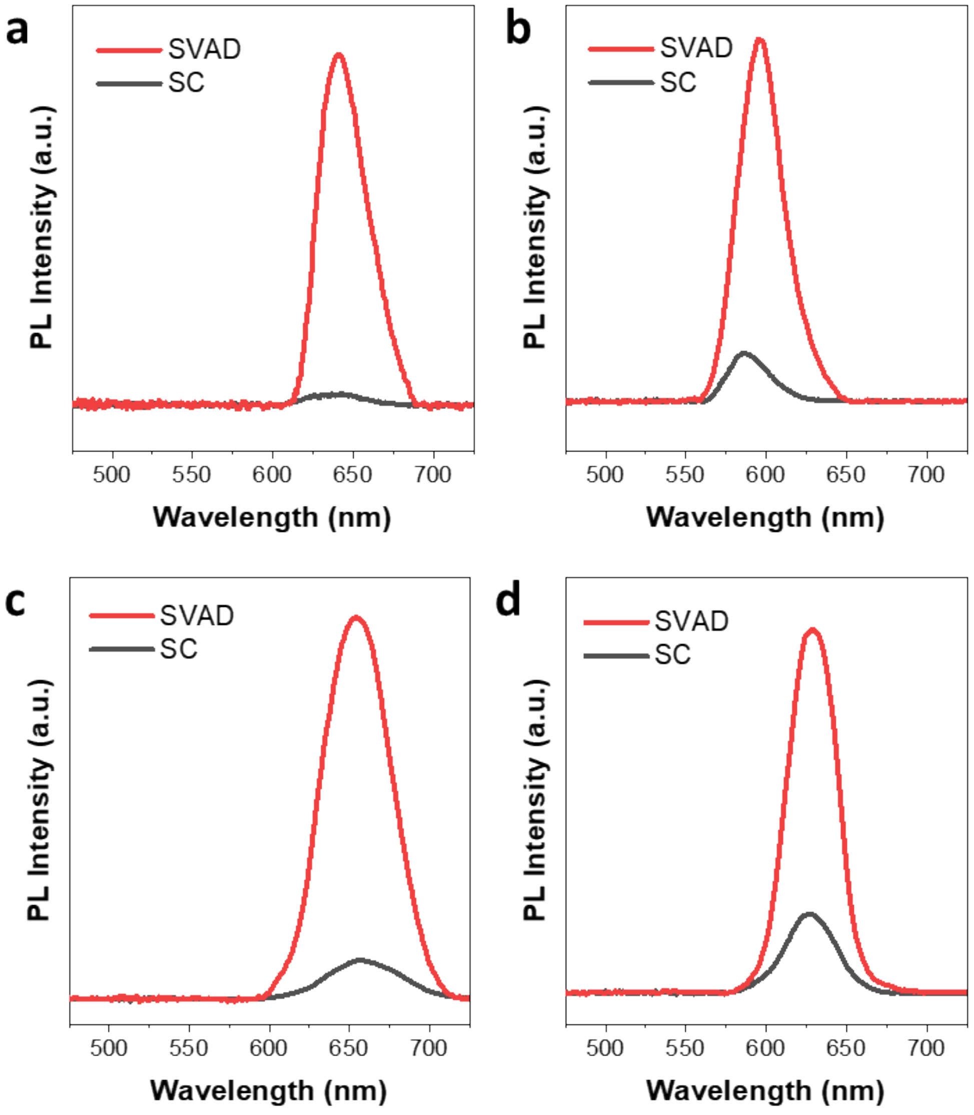

Figure S10. Steady-state photoluminescence (PL) spectra for perovskite films of (a) (DMePDA)SnI_4, (b) (HDA)SnI_4, (c) (CDMA)SnI_4, and (d) (PDMA)SnI_4.

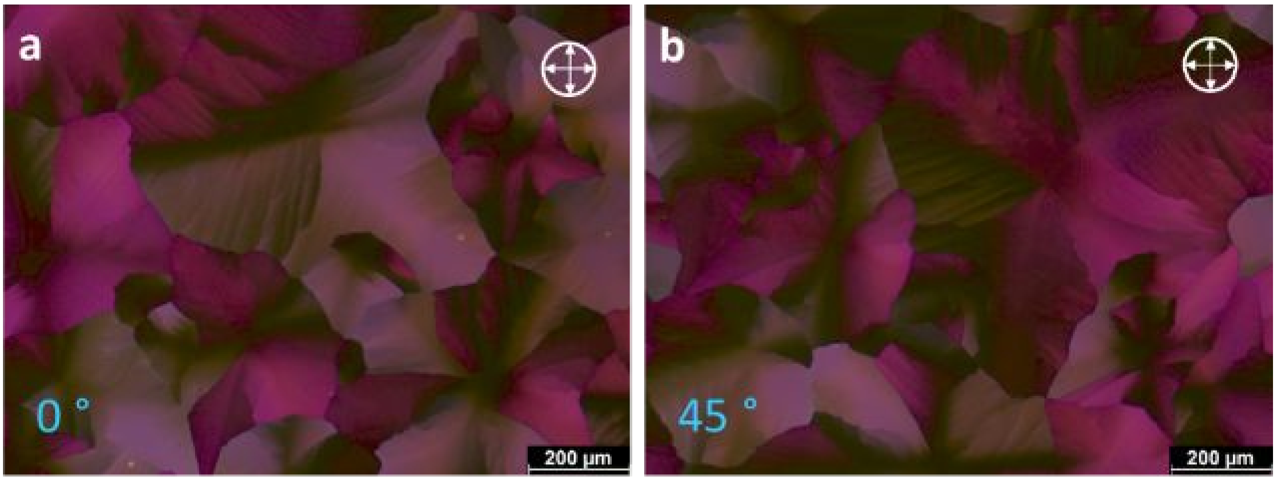

Figure S11. Polarized optical microscope for SVAD (PDMA)SnI_4 film at (a) 0° and (b) 45°.

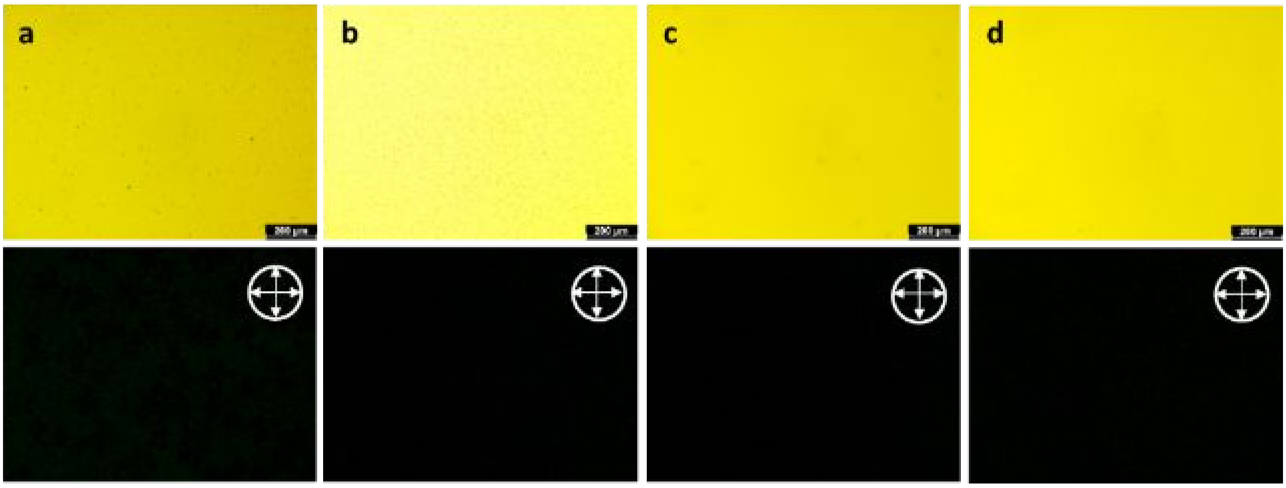

Figure S12. Optical microscope (upper) and polarized optical microscope (below) for SC films of (a) (DMePDA)SnI_4, (b) (HDA)SnI_4, (c) (CDMA)SnI_4 and (d) (PDMA)SnI_4.

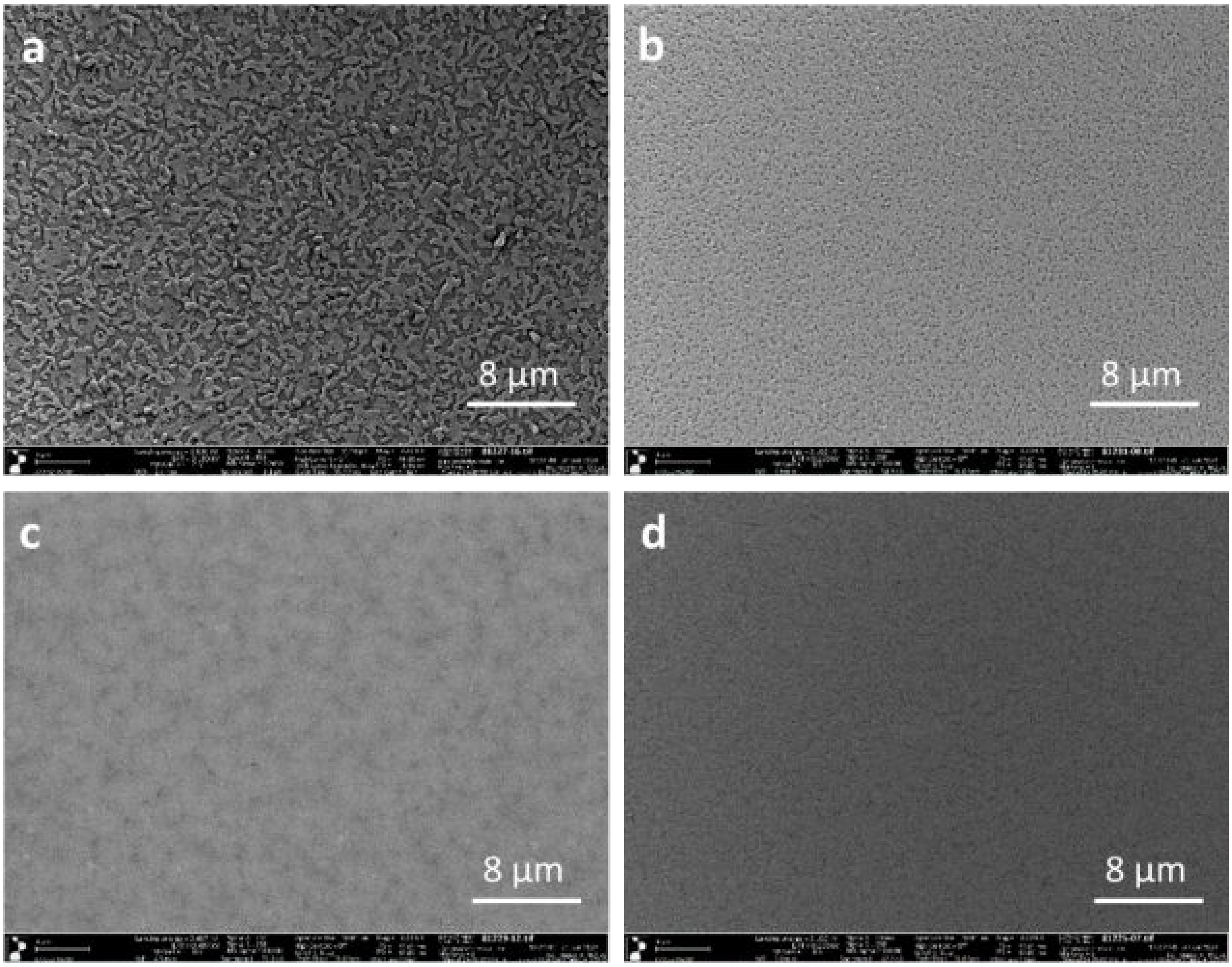

Figure S13. Scanning electron microscope for SC films of (a) (DMePDA)SnI_4, (b) (HDA)SnI_4, (c) (CDMA)SnI_4 and (d) (PDMA)SnI_4 .

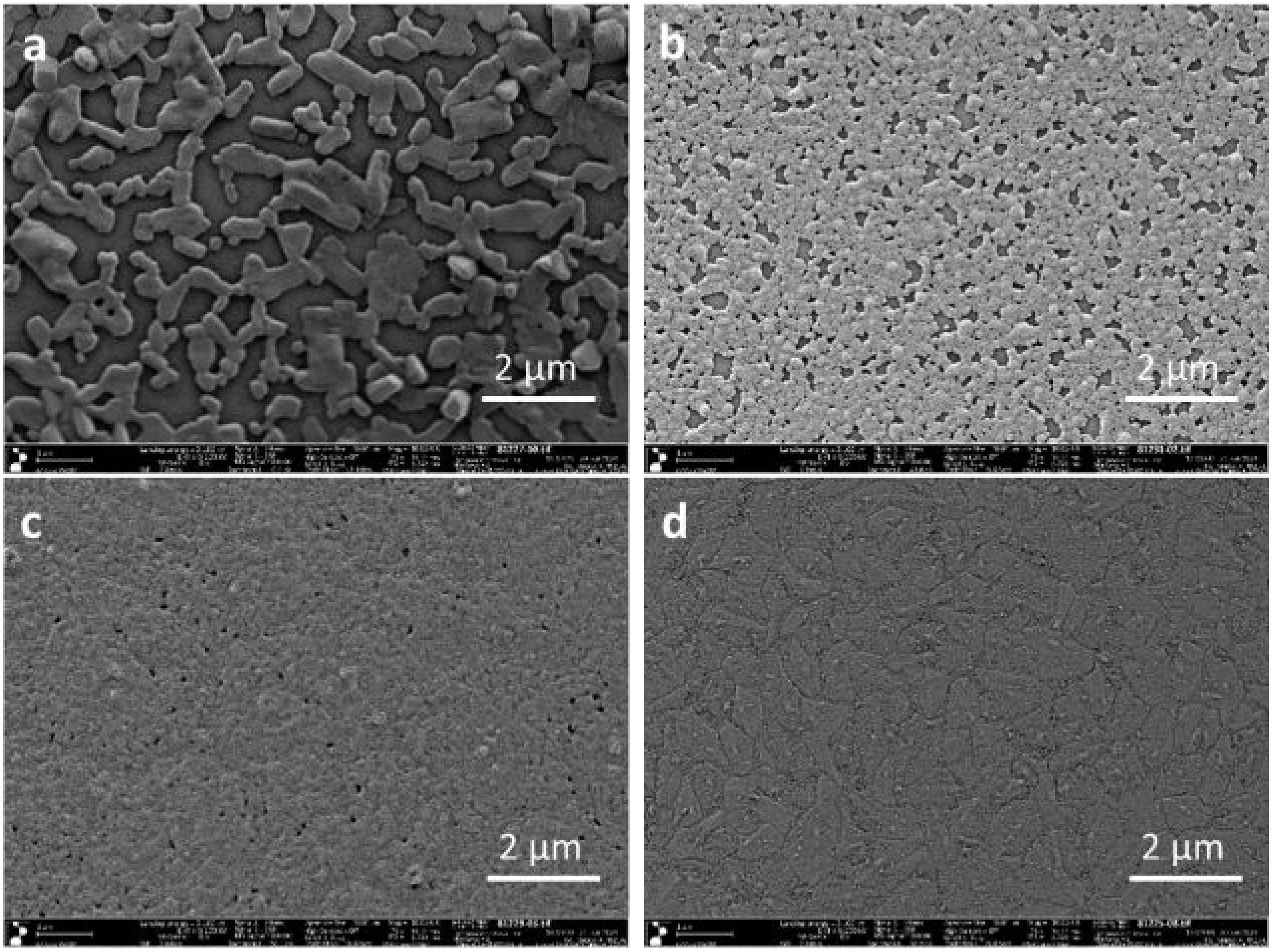

Figure S14. Scanning electron microscope for SC films of (a) $(DMePDA)SnI_4$, (b) $(HDA)SnI_4$, (c) $(CDMA)SnI_4$ and (d) $(PDMA)SnI_4$ with the scale of 2 μm.

Table S3. Local charge carrier mobilities of SC and SVAD DJ perovskites derived from Figure 4a.

**Processing**	**$(DMePDA)SnI_4$**	**$(HDA)SnI_4$**	**$(CDMA)SnI_4$**	**$(PDMA)SnI_4$**
SVAD ($cm^2\ V^{-1}\ s^{-1}$)	1.1	1.8	3.2	3.4
SC ($cm^2\ V^{-1}\ s^{-1}$)	0.6	1.1	1.8	1.6

Table S4. Characterization of perovskite FETs processed by SVAD and SC.

		$(DMePDA)SnI_4$	$(HDA)SnI_4$	$(CDMA)SnI_4$	$(PDMA)SnI_4$
SVAD	μ_{FET} ($cm^2 V^{-1} s^{-1}$)	1.6×10^{-3}	5.1×10^{-3}	0.02	0.18
	V_{TH} (V)	18.2	4.7	13.5	5.1
	SS ($V dec^{-1}$)	9.1	8.3	9.1	3.9
SC	μ_{FET} ($cm^2 V^{-1} s^{-1}$)	-	2.5×10^{-4}	4.5×10^{-4}	2.6×10^{-3}
	V_{TH} (V)	-	4.7	6.8	11.7
	SS ($V dec^{-1}$)	-	38.5	33.4	15.6

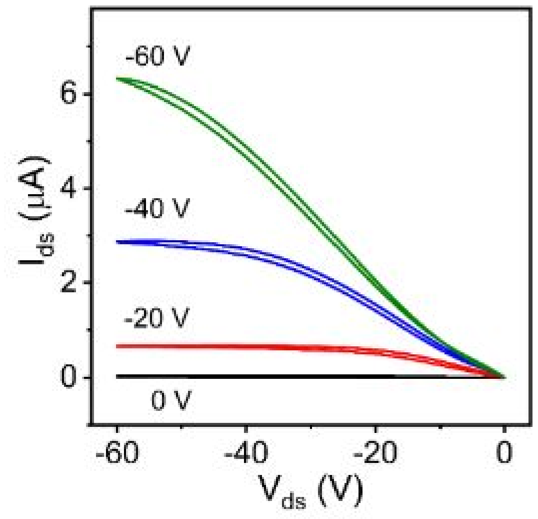

Figure S15. Output characteristics of SC $(PDMA)SnI_4$ FET.
